# Monitoring the Spread of Swine Enteric Coronavirus Diseases in the United States in the Absence of a Regulatory Framework

**DOI:** 10.3389/fvets.2016.00018

**Published:** 2016-03-14

**Authors:** Andres M. Perez, Anna Alba, Dane Goede, Brian McCluskey, Robert Morrison

**Affiliations:** ^1^Department of Veterinary Population Medicine, College of Veterinary Medicine, University of Minnesota, St. Paul, MN, USA; ^2^Veterinary Services, Animal and Plant Health Inspection Service, U.S. Department of Agriculture, Fort Collins, CO, USA

**Keywords:** swine enteric coronavirus, epidemiology, monitoring, United States

## Abstract

The reporting and monitoring of swine enteric coronavirus diseases (SECD), including porcine epidemic diarrhea virus and porcine delta coronavirus, in the United States have been challenging because of the initial absence of a regulatory framework and the emerging nature of these diseases. The National Animal Health Laboratory Network, the Emergency Management and Response System, and the Swine Health Monitoring Project were used to monitor the disease situation between May 2013 and March 2015. Important differences existed between and among them in terms of nature and extent of reporting. Here, we assess the implementation of these systems from different perspectives, including a description and comparison of collected data, disease metrics, usefulness, simplicity, flexibility, acceptability, representativeness, timeliness, and stability. This assessment demonstrates the limitations that the absence of premises identification imposes on certain animal health surveillance and response databases, and the importance of federally regulated frameworks in collecting accurate information in a timely manner. This study also demonstrates the value that the voluntary and producer-organized systems may have in monitoring emerging diseases. The results from all three data sources help to establish the baseline information on SECD epidemiological dynamics after almost 3 years of disease occurrence in the country.

## Introduction

Since the first detection of porcine epidemic diarrhea virus (PEDV) in the United States (US) in May 2013, followed by the subsequent detection of porcine delta coronavirus (PDCoV), many efforts have been made to monitor the spread of swine enteric coronavirus diseases (SECD) in the country (1–7). PEDV and PDCoV reporting to the World Organization for Animal Health (OIE) are not mandatory, although the reporting of SECD infection is encouraged due to their emerging nature and important economic impact on swine industry (8).

To investigate the temporal and spatial spread of SECD in the US and to obtain information to support decision-making, the US Department of Agriculture (USDA) used three data sources, namely, the National Animal Health Laboratory Network (NAHLN) (9–11), the Emergency Management and Response System (EMRS) (12), and the Swine Health Monitoring Project (SHMP) (13). These sources differed on a number of qualitative and quantitative attributes, such as the target population, the temporal extent of the collected data, the case definition, and the nature of reporting.

The goal of this report is to report the attributes and conduct a critical assessment of the three systems used to monitor the spread of SECD in the US between May 2013 and March 2015. Their evaluation is approached from different perspectives. First, we describe features associated with each dataset and their respective attributes. Second, the databases are compared in terms of shape and size of the epidemic curves generated from the data in each. Finally, different qualitative aspects (usefulness, simplicity, flexibility, quality of data, acceptability, representativeness, and timeliness and stability) are described and compared in order to identify strengths and weaknesses and assess their potential application to the surveillance for SECD. The report here contributes to understand the nature and extent of the data collected to monitor PEDV and PDCoV in the US and helps to establish the baseline information on the SECD epidemiological dynamics after 2 years of disease spread in the country.

## Materials and Methods

### Background of Data Sources

#### National Animal Health Laboratory Network

The NAHLN is a coordinated network of federal and state institutions, which includes universities and animal disease diagnostic laboratories that collaborate to provide diagnostic testing to animal health surveillance and give response to important disease events (9–11). The NAHLN laboratories provided weekly data files of the PEDV-PCR test result records (14, 15), including results from each sample tested and, if available, the associated data on the collection site, state, and animal age.

A laboratory accession with one or more PEDV PCR positive samples was considered positive. In most of the cases, NAHLN data did not include information to identify individual premises or herds.

**Figure 1:**
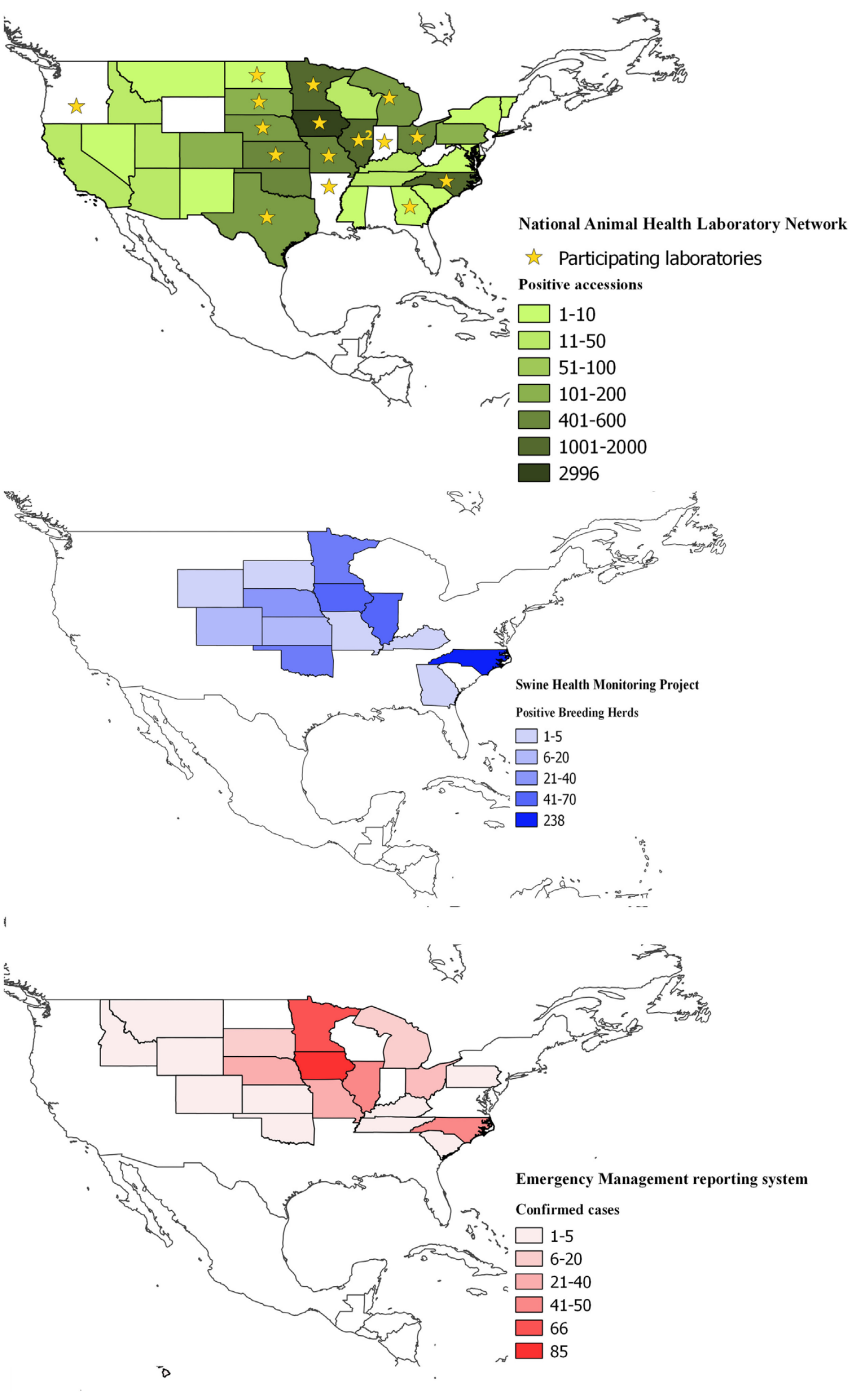
**Spatial coverage of the three information systems used to collect data on enteric coronavirus records in the United States between May 2013 and March 2015; top (in green), the National Animal Health Laboratory Network (NAHLN); middle (in blue), Swine Health Monitoring and bottom (in red), Emergency Management Reporting System**. Shades depict the number of reports on each database and state. Stars in the NAHLN figure indicate the location of the participating laboratories, including Arkansas Livestock and Poultry Commission-Veterinary Diagnostic Laboratory; Athens Veterinary Diagnostic Laboratory, University of Georgia; University of Illinois Veterinary Diagnostic Laboratory; Illinois Department of Agriculture, Galesburg Animal Disease Laboratory; Iowa State University Veterinary Diagnostic Laboratory; Kansas State Veterinary Diagnostic Laboratory; Michigan State University–Diagnostic Center for Population and Animal Health; University of Minnesota Veterinary Diagnostic Laboratory; Veterinary Medical Diagnostic Laboratory, University of Missouri; University of Nebraska Veterinary Diagnostic Center; USDA National Veterinary Services Laboratories; Rollins Diagnostic Laboratory, North Carolina Department of Agriculture; Veterinary Diagnostic Laboratory, North Dakota State University; Ohio Animal Disease Diagnostic Laboratory–Ohio Department of Agriculture; Oregon State University Veterinary Diagnostic Laboratory; Indiana Animal Disease Diagnostic Laboratory, Purdue University; Animal Disease Research and Diagnostic Laboratory, South Dakota State University; and Texas Veterinary Medical Diagnostic Laboratory, Texas A&M University.

The coverage of the NAHLN data was that of the participating laboratory’s service areas. As of May 2014, 24 veterinary diagnostic laboratories voluntarily reported PEDV testing data to the USDA NAHLN program office (Figure 1).

#### USDA Emergency Management and Response System

The USDA EMRS is an information technology system designed to provide timely and effective response to animal health emergencies, including foreign animal disease (FAD) investigations and state and national animal disease incidents (12). Federal, state, and tribal animal health officials use the EMRS to record and view animal disease data, manage incident response services and resources, and create reports and maps to facilitate disease investigations and associated epidemiological analyses. On June 5, 2014, according to the Federal Order (FO) issued by the USDA, the EMRS was designated as the official system for the recording of all SECD situation data collection, information management, and reporting. The FO mandated the reporting of SECD cases (16). The EMRS also received testing data provided by the NAHLN laboratories. The NAHLN data were electronically transmitted into the EMRS to facilitate the response activities. The emergency response officials investigated all the SECD case reports that were initiated by sharing the lab results or other communication channels and determined the SECD status of each reported herd/premise. On the basis of lab test results and consultations with herd owners and herd veterinarians, the premises received a status of confirmed or presumptive positive for PEDV, PDCoV, or both viruses. In accordance with the USDA case definition, a confirmed positive herd/premise was a site with animals that had at least one positive for PEDV or PDCoV to a PCR test and a history of clinical signs consistent with SECD, whereas a presumptive herd/premise had animals that tested positive for either disease without manifesting clinical signs (17, 18). The status of the premises was reported into the EMRS as soon as a federal or state official had confirmed all the information sources required and the status could be updated, modified, or closed until the new information became available.

#### Swine Health Monitoring Project

This system was designed by the University of Minnesota in collaboration with the American Association of Swine Veterinarians and the National Pork Board and was aimed at monitoring important diseases that affected the swine industry. Initially, the SHMP included data of porcine reproductive and respiratory syndrome (PRRS) cases collected since July 17, 2013. When the PEDV epidemic started, the infrastructure of the SHMP was used to collect SECD incidence data. The project collects data routinely from breeding farms (including commercial/multiplier/nucleus). The participation in the project and the contribution to the database on a weekly basis was voluntary (13). The attributes collected by this system included the premises ID, the coordinates, the state and county of herd, the average inventory/capacity, the type of breeding herd, the air filtration status, and the location of nearby pig farms (<3 miles). The individual data were informed to the participants of the study.

From the data gathered by each source, we described and compared the respective epidemic curves.

### Evaluation of the SEDC Monitoring Systems from Different Perspectives

Based on the guidelines proposed by Salman et al. (19, 20), we assessed the following aspects of each system:

1. usefulness (contribution to the prevention and control of diseases),
2. simplicity (considering the operability and logistics),
3. flexibility (ability to adapt to changing information needs or operating conditions),
4. quality of data (completeness and validity of the data recorded),
5. acceptability (willingness of people and organization to participate),
6. representativeness (cases detected by the system that represent the true situation),
7. timeliness (speed between steps in a surveillance system and providing feed-back information), and
8. stability (ability to function without failure and availability when the system is needed).

Although the evaluation of sensitivity and positive predictive value were not under the scope of this study, since it would require other quantitative approaches, this work also provided some evidences from the analysis of outcomes.

## Results

### Comparison of Basic Features and Description and Comparison of Epidemic Curves from Data Gathered by Each System

**Figure 2:**
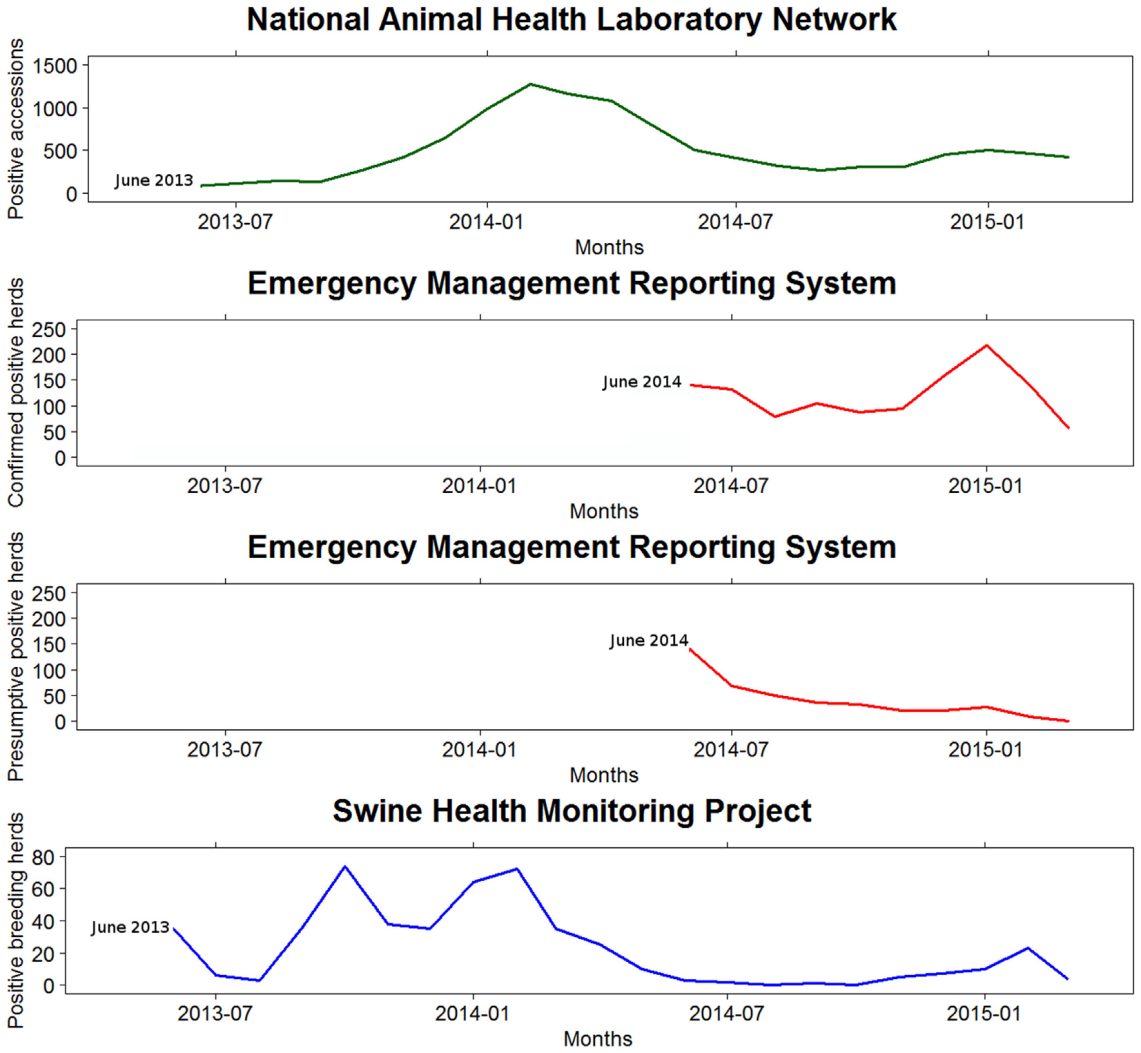
**Monthly swine enteric coronavirus records reported in the United States by the three monitoring systems between May 2013 and March 2015**.

There were substantial differences between and among the three information systems used to monitor the SECD spread in the US (Table 1). Hence, the epidemic curves built from the monthly SECD records reported by the three monitoring systems showed different shapes (Figure 2).

**Table 1:** **Comparative features associated with the National Animal Health Laboratory Network (NAHL), Emergency Management and Response System (EMRS), and Swine Health Monitoring Project (SHMP) databases**.

Database	National Animal Health Laboratory Network (NAHLN)	Emergency Management and Response System (EMRS)	Swine Health Monitoring Project (SHMP)
Purpose	Compilation of PEDV PCR testing data for analysis, reporting, and decision support	Occurrence of PEDV in the swine herds/premises	Situational awareness for the participating systems
		Tracking of disease response and control actions	
Start date	June 16, 2013	June 5, 2014	May 13, 2013
Unit record	Test result	Premise/herd	Breeding herd
Coverage	National (all sites)	National (all sites)	Participating systems
Case definition	A sample positive to PEDV PCR	Confirmed herd: at least one pig positive to PEDV PCR plus pigs with clinical signs	Farm in which PEDV was reported by the veterinarian, based on clinical signs and diagnostic test results
		Presumptive herd: at least one pig positive to PEDV PCR and no clinical signs observed	
Frequency of data submissions	Weekly until September 2014. After, some daily submissions using the HL7 electronic messaging	Daily	Weekly
Participation	Voluntary NAHLN labs	Mandatory according to the federal ordering	Voluntary producers of breeding herds
Access to available data	USDA staff and lab participants	State and Federal animal health officials	Public: as aggregated data
			Participants: raw and de-identified data
Reporting	Weekly reports of amount of positive and negative accessions by week, month, and state publicly available	Weekly reports; summaries of the positive premises by week, month, and state; reports available to the public	Weekly reports depicting time series for participating systems

These differences could be explained, at least in part, to diverse reasons related to the implementation of each system. The SHMP was available from the beginning of the epidemic and essential to assess the initial stages, since the reporting of SECD from NAHLN or EMRS was not in place at that moment. The SHMP data were spread over an extended period of time, suggesting a lower transmission rate compared to what may be estimated using the EMRS data – noteworthy, NAHLN data would not allow estimating a transmission rate. In the NAHLN and EMRS, many reports were concentrated at the beginning of the curve, likely, because they included a number of reports that occurred earlier in the epidemic. In contrast, in the winter of 2014–2015, when the three databases had been in place for almost 2 years, all the epidemic curves pointed toward an increase of PEDV incidence versus the previous summer levels, which is particularly evident from data in the EMRS database. Although the magnitude of the peak differed between databases, the epidemic curves reflected an upward trend of PEDV from October 2014 until January 2015.

**Figure 3:**
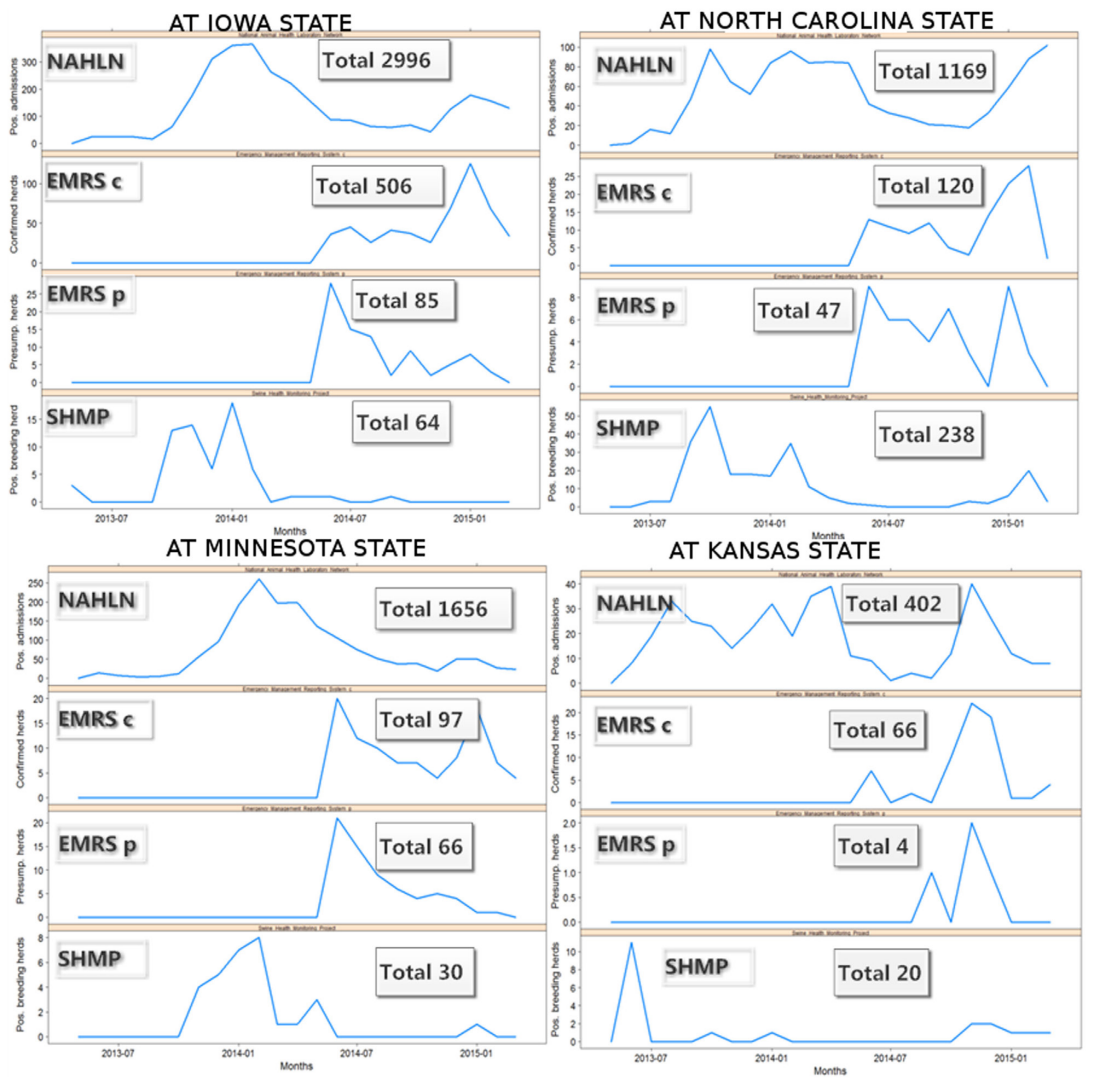
**Monthly swine enteric coronavirus records in the four US States with more positive herds reported between May 2013 and March 2015**.

Because one single state (Iowa) accounts for almost 50% of the swine farms in the country, the epidemic curves were also explored at state level to assess if the pattern observed in Iowa dominated the evidence for the entire country, or if a similar pattern was observed at the state level, for those states that contribute to most of the swine production in the country. Although the disease dynamics seemed to be slightly different between states, probably associated with different times of disease introduction, demographic, and epidemiological conditions; the most swine-densely populated states showed similar trends over time, suggesting that the pattern observed in the entire country is not just a reflection of what occurred in Iowa (Figure 3).

### Evaluation of the SECD Monitoring Systems

#### Usefulness

The NAHLN database provided information of almost every single SECD diagnosis in the US. This information was crucial to determine which SECD viruses were circulating. However, this system was not sufficient to determine the main measures of disease frequency or for decision-making, since the individual identification of infected premises or herds was not recorded.

In contrast to NAHLN, the EMRS database comprised complete and reliable data at the farm level for epidemiological purposes, including all farm types, and containing data for positive farms. When the EMRS was in place, the SECD occurrence and spread could be investigated and the system was used to determine prevention and control measures.

The SHMP served to assess the initial stages of the epidemic, when most of the farms were infected. The SHMP data referred only to sow farms from some systems and could not be inferred to the entire country, but this system helped to create the foundations for an ongoing monitoring system database for emerging and non-reportable diseases in the US.

#### Simplicity

The USDA–NAHLN asked to volunteer labs and universities their SEDC testing data and share this information electronically. This system followed by NAHLN was much simpler and cheaper than the EMRS.

The EMRS had to collect and verify all the data received at the premise level and these tasks required extensive field work by USDA staff.

The SHMP asked to volunteer swine producers of breeding herds and only operated in some states. Logistics of implementation were simpler than both NAHLN and EMRS.

#### Flexibility

The NAHLN demonstrated the capacity to adapt as a result of the legal requirements of the FO on June 5, 2014. First, a new Laboratory Messaging System (LMS), data management system, was launched to store and manage all PEDV testing data. This new data system was the USDA’s repository for laboratory testing data and was connected to other key data systems, including EMRS. The LMS provided a system to transmit testing data electronically to USDA by using HL7 electronic result messaging technology. As of March 2015, five NAHLN laboratories were electronically messaging their PEDV test results to USDA rather than providing weekly data files. Another major change included the requirement of the premises identification data with the laboratory records. However, even with this requirement, the PEDV laboratory test records often did not include the premises identifiers, since the individual lab policies or the premises information were not provided by the submitters. The inclusion of the national premises identification data with the testing records slowly increased from 40% in June 2014 to nearly 90% in October 2014. In terms of flexibility, logistics for implementation of the EMRS was more complex, and consequently, its adaptation to new situations required more time and effort than the NAHLN or the SHMP.

#### Quality of Data

The labs that participated in the NAHLN had been previously certified for testing samples to confirm SECD diagnosis.

The testing data of NAHLN did not record the premises identification and prevented USDA from knowing exactly how many farms or premises were infected. The lack of this attribute was a major constraint in using the NAHLN database for decision-making. The location of herds could be roughly determined by the Collection Site State variable reported by the lab, but unfortunately, this information was not completely reliable. The labs could not validate the location data and the state location could correspond to the corporate headquarters, the submitting veterinarian, or the billing address. The EMRS was specifically designed to enable accurate determination of the number and location of infected herds. In the EMRS, the PEDV-positive premises were confirmed, whereas the laboratory results data reported by the NAHLN labs were not confirmed. The participation of SHMP was voluntary and provided complete information of important disease at farm level. This information could not be checked in the field, but one would expect a minimum impact of biases associated with the reluctance of information sharing.

#### Acceptability

The participation was voluntary in both SHMP and NAHLN.In the SHMP numerous swine producers voluntarily agreed to share their data from their breeding farms.The effort of NAHLN was unprecedented and increased from 5 laboratories in June 2013 to 18 laboratories by May 2014. Moreover, to encourage the participation, on June 5, 2014, with the FO, the NAHLN laboratories were reimbursed for SECD testing costs by the USDA, and these savings passed along to individual producers and companies.

The EMRS required the reporting of all cases of novel SECD to USDA or State animal health officials under a federally regulated framework.

#### Representativeness

In the NAHLN, 18 out of 24 diagnostic laboratories shared SECD testing data. All major veterinary diagnostic laboratories in the key swine-producing areas of the US Service participated. These laboratories covered 41 states plus Puerto Rico and the vast majority of US swine-production facilities. Over time, the number of lab accessions and reporting of NAHLN data improved. Although the reasons why some laboratories have not shared information are unclear, it is possible that they may simply not have had SECD cases to report or tests conducted.

In spring of 2013, the NAHLN laboratories began to voluntarily share the PEDV testing information in an effort to assess and understand the emerging PEDV situation. In June 2013, at the request of the laboratories, the NAHLN of the USDA program office started facilitating the aggregation and reporting of PEDV testing data to offer national level information. From June to October 2013, only the positive result records were shared; after November 1, 2013, the labs provided both positive and negative result records.

During the spring of 2014, the NAHLN labs started to share PDCoV testing data in addition to PEDV testing data. In May 2014, the NAHLN labs collectively provided over 6,000 PEDV-PCR test records per week and the number of PEDV-positive laboratory accessions increased from <50/week to more than 300. As of March 2015, the NAHLN laboratories had shared PEDV results from over 30,000 lab accessions, including data of 3,880 positive accessions.

The EMRS included 1,616 premises records associated with the SECD situation, including 1,210 PEDV confirmed-positive premises and 406 PEDV presumptive-positive premises as of March 2015. This system was mandatory for all the country.

This SHMP database covered 752 breeding farms (including commercial/multiplier/nucleus) and in March 2015 this source represented approximately 2,110,000 sows (out of a national total of approximately 5.8 million). The information was supplied by 23 production systems distributed in 16 states. The total cases were 467 as of March 2015 (i.e., 28.9% of cases compared to the 1,616 SEDC cases confirmed by EMRS).

#### Timeliness and Stability

The USDA–NAHLN compiled the data into a standardized dataset for analysis and distributed weekly reports summarizing the PEDV laboratory testing information.

The EMRS compiled the data daily and reported the extracted information by week. This system also provided summaries of the positive premises by week, month, and state. These reports were available to the public.

The SHMP database operated using weekly reporting updates with quarterly review of reports to ensure its accuracy. All the aggregated data were available to the public in the form of weekly updated charts showing the estimates of cumulative incidence, status prevalence, and weekly incidence trends (though raw data were not distributed).

The participation in both NAHLN and SHMP was voluntary, and thus the collaborators could terminate at any time, whereas the EMRS was mandatory. However, the unique program available at the beginning of the epidemic was the SHMP, followed by the NAHLN, and finally by the EMRS.

Outcomes evidenced that the leverage of multiple data sources enhanced the capacity to detect positive cases over time, and the EMRS provided the most reliable status of infection at herd/premise level. It should be noted that each system uniquely had its own raw data, being available to the others for only review and discussion.

## Discussion

The monitoring of the SECD progress in the US between May 2013 and March 2015 supposed an important challenge due to the emerging nature of these viruses and the absence of a regulatory framework. The three monitoring systems described in this work allowed getting useful information for supporting decision-making and defining actions in government or industry sectors. However, important differences existed between and among them in terms of nature and extent of reporting, with both strengths and weaknesses.

An important advantage of the NAHLN database was that one may expect that almost every single SECD diagnosis in the US was included there. However, a major drawback of the NAHLN database was the absence of unique premises identification numbers (PINs), which limited the interpretation of results to individual samples. Although this system illustrated the explosive increase in samples testing positive for the disease, the NAHLN data could not be used to answer basic questions about the number or the location of infected premises. This limitation would be easily overcome by promoting the inclusion of PINs in laboratory sample submissions, although such implementation would require active education of US practitioners on the importance of recording such information on their submission forms (21).

The mandatory reporting of disease, which included PINs and other basic epidemiologic data with maintenance in the EMRS database, facilitated to get most accurate estimates of disease incidence or prevalence and geographic distribution (7). Unfortunately, the FO that regulated the official reporting of cases came too late to allow the follow-up of the disease spread and its scope in the critical early stages of the outbreak.

Regarding the SHMP, this system was the first to be put in place, and for this reason, the information collected in this database was critical to assess the initial stages of the epidemic, when most of the farms were infected. Additionally, because the participants voluntarily agreed to share their data, one would expect a minimum impact of biases associated with the reluctance of information sharing. However, the SHMP data referred only to sow farms and the information extracted are not representative of the situation throughout the country. The SHMP database demonstrated that the US swine industry had the potential to self-regulate the reporting of cases. But the potential caveat was that, being a voluntary system, the database could be biased and the sensitivity of reporting could vary spatially and temporally. However, the information routinely collected by the SHMP may well serve to create the foundations for an ongoing monitoring system database in place for monitoring emerging and non-reportable diseases in the US.

In conclusion, in the absence of a regulatory framework at the beginning of the epidemic, leveraging of multiple data sources of different nature and range of reporting allowed to monitor the spread of SEDC through the US swine herd. Results demonstrate the importance of collecting individual farm information in order to produce accurate estimates of disease spread. Experience gained from the case epidemic reported here may be useful to monitor future FAD incursions in the US.

## Author Contributions

AP, BM, and RM planned the study. BM was primarily responsible for the analysis of two of the databases, whereas AA-C and DG analyzed the remaining database. AP and RM wrote most of the paper, whereas other authors contributed significantly to the writing.

## Conflict of Interest Statement

The authors declare that the research was conducted in the absence of any commercial or financial relationships that could be construed as a potential conflict of interest.
