# Corrigendum: Monitoring the Spread of Swine Enteric Coronavirus Diseases in the United States in the Absence of a Regulatory Framework

**DOI:** 10.3389/fvets.2021.794591

**Published:** 2021-11-10

**Authors:** Andres M. Perez, Anna Alba, Dane Goede, Brian McCluskey, Robert Morrison

**Affiliations:** ^1^Department of Veterinary Population Medicine, College of Veterinary Medicine, University of Minnesota, St. Paul, MN, United States; ^2^Veterinary Services, Animal and Plant Health Inspection Service, U.S. Department of Agriculture, Fort Collins, CO, United States

**Keywords:** swine enteric coronavirus, epidemiology, monitoring, United States

In the original article, the **Conflict of Interest** statement was incomplete. The statement appears below.

## Conflict of Interest

“The handling editor BM-L and the reviewer LM declared a past co-authorship with one of the authors AP and states that the process nevertheless met the standards of a fair and objective review.”

The authors apologize for this error and state that this does not change the scientific conclusions of the article in any way.

## Publisher's Note

All claims expressed in this article are solely those of the authors and do not necessarily represent those of their affiliated organizations, or those of the publisher, the editors and the reviewers. Any product that may be evaluated in this article, or claim that may be made by its manufacturer, is not guaranteed or endorsed by the publisher.

